# Resorcin

**Published:** 1888-01

**Authors:** Francis H. Williams


### *RESORCIN.*

This drug is favorably reported in the treatment of cutaneous and gastric disorders. Dr. Edward Maclay, in *London Medical Record*, claims for it the antiseptic properties of carbolic acid, without the irritant properties of that substance. A two per cent. solution is antiseptic, and unirritating to the tissues. It seems to possess special advantages in the treatment of gastric and cutaneous disorders. Dr. Maclay reports two cases of gastric ulcer, with immediate recovery under its use. In gastric catarrh, not relieved by bismuth, soda, etc., recovery followed at once under treatment with resorcin. Intestinal catarrhs, chronic diarrhœa, etc., were in no way benefitted by resorcin, nor were the evacuations disinfected, constituting a marked contrast to the actions of naphthalin, and suggesting a special field of action for each of these two drugs: naphthalin for intestinal disorders, or where-ever it is desirable to disinfect the intestinal canal, or render antiseptic its contents; resorcin for the same purpose in the stomach, and externally in sensitive tissues. Internally, Dr. Maclay employs five-grain doses of resorcin, three times a day, given in water with a little glyce rine, or in chloroform water. The taste is peculiar, but slight and easily tolerated. Much larger doses have been employed, but are not advisable, thirty grains having caused febrile disturbance, and sixty grains marked toxic action, dizziness and collapse.—*Francis H. Williams, M. D., in Medical and Surgical Journal.*

